# Pretreatments for enhancing sewage sludge reduction and reuse in lipid production

**DOI:** 10.1186/s13068-020-01844-3

**Published:** 2020-12-14

**Authors:** Jiaxin Chen, Ji Li, Xiaolei Zhang, Zhaoyang Wu

**Affiliations:** 1 Department of Civil and Environmental Engineering, Shantou University, 243, Daxue road, Shantou, 515063 Guangdong People’s Republic of China; 2 Department of Civil and Environmental Engineering, Harbin Institute of Technology, Shenzhen, People’s Republic of China

**Keywords:** Sludge reduction, Lipid production, Pretreatment, Ultrasonication, Biodiesel

## Abstract

**Background:**

Converting wastewater sludge to lipid is considered as one of the best strategies of sludge management. The current problem of lipid production from wastewater sludge is the low yield (0.10–0.16 g lipid/g dry sludge) due to the low availability of easily uptaken materials (such as soluble monosaccharide and oligosaccharide) in sludge to oleaginous microorganism (*Rhodotorula glutinis*, *Trichosporon oleaginosus*, *Lipomyces starkeyi*). Pretreatments are efficient methods to improve sludge bioavailability. This study is aimed to achieve high lipid production from sludge and high sludge reduction.

**Results:**

In this study, it was observed that the soluble chemical oxygen demand (SCOD) had significantly increased after different pretreatment. The SCOD in the supernatant was increased from 32.64 to 180.25 mg/L, 924.16 mg/L, 1029.89 mg/L and 3708.31 mg/L after acidic (pH 2 for 2 h), alkaline (pH 12 for 2 h), microwave irradiation (15 min with 5 min interval), and ultrasonication (30 min at 450 W and 20 kHz frequency with 5 s on and 2 s off mode) pretreatment, respectively. Pretreatments have also increased the release of total nitrogen (TN) and total phosphorus (TP) from solids. The sludge after different pretreatments were used as a medium for lipid production, and the highest lipid content (36.67% g/g) was obtained in the fermentation with ultrasonication pretreatment sludge, and the sludge reduction was 63.10%. For other pretreatments, the lipid content and sludge reduction were 18.42% and 32.63% in acid pretreatment case, 21.08% and 36.44% in alkaline pretreatment case, and 26.31% and 43.03% in microwave pretreatment case, respectively.

**Conclusion:**

It was found that ultrasonication pretreatment was the most efficient way to increase the sludge biodegradability (SCOD) and to release TN and TP from solid phase to liquid phase. Pretreated sludge for lipid production achieved significant improvement in lipid yield and sludge reduction. Lipids produced from pretreated sludge were transesterified to biodiesel and the analysis showed that biodiesel had a similar composition as commercial biodiesel. The study reveals that pretreatment on sludge is a promising method for enhancing biological sludge management efficiency.
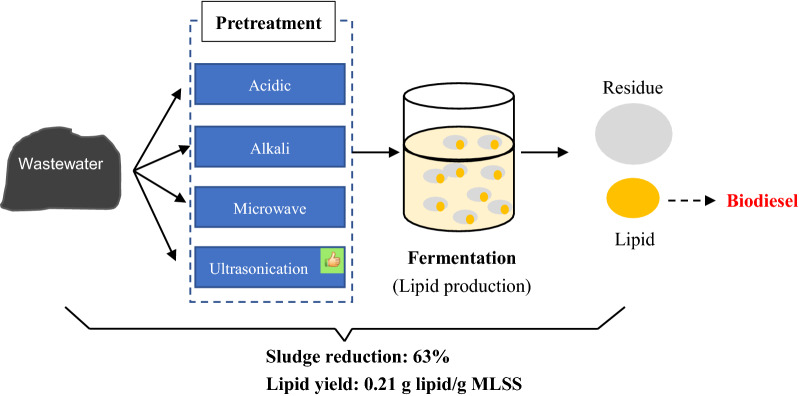

## Background

Along with the development of society, wastewater discharge amount sharply increases due to human activities [14]. For instance, it was 26.10 billion tonnes in 2004 and dramatically increased to 51.00 billion tonnes in 2014 in China [62]. Aiming to reduce its environmental and health risks, wastewater was collected and mainly treated by activated sludge process, biofilm process, or membrane bioreactor process in the wastewater treatment plants. Nevertheless, a large amount of sewage sludge (5–8 tonnes sludge with 80% water content is generated in every 10,000 tonnes wastewater treated), which is an unavoidable by-product of wastewater treatment, is generated [14]. For decades, sewage sludge was considered as a waste. Treatments, such as digestions (aerobic and anaerobic), landfill, and incineration, are mainly used for the reduction or disposal of sewage sludge [51, 56]; however, digestions and landfill require large land, and incineration causes high energy and cost input [37, 38].

It has been well realized that sewage sludge contains various useful materials such as carbon, nitrogen and phosphorus, which can be recovered by physical, chemical and/or biological methods [56]. Recovery of nitrogen and phosphorus as fertilizers is one of the best choices [4]. Carbon recovery from sludge should be given the main focus as it is the most abundant component in sewage sludge [5, 13]. Carbon recovery from sludge is generally accomplished through direct extraction or converting it to value-added products [13]. Biogas, lipids, extracellular polymeric substances, bioplastics, short-chain fatty acids are common value-added products generated from sewage sludge [34, 39, 63]. It provides new option for sludge management and resource recycling [1].

Lipids are good sources for biodiesel production [63]. It has been found both in the primary and secondary sludge; however, the lipid content in raw sludge (g lipids/g sludge) is quite low [59]. Thus, direct lipid extraction from sludge has limited sludge reduction and is not attractive [45]. Refermentation of sludge is a promising way to increase lipid content and enhance sludge reduction [40]. Additionally, it was found that biodiesel production from lipid accumulated by sludge fermentation had economic and energetic feasibility, and it was principally depending on the amount of sludge reduction and lipid accumulation [6, 60].

Raw sewage sludge is a mixture of complex organic compounds including protein, carbohydrates, and lipid [52], which has very low biodegradability. Pretreatment is an efficient way of breaking down complex materials in sludge [3, 61]. Physical, chemical, and biological pretreatments were investigated to release nutrients from sewage sludge and were observed to highly assist the utilization of sludge by microorganisms [3]. So far, the impact of sludge pretreatment on methane and hydrogen production has been widely performed [10, 25, 27, 32, 47, 50, 64], however, few have evaluated its impact on lipid production [25, 27, 42]. Due to the concern of high cost, difficult management, and low stability, the full-scale application of biological pretreatments is scarce [2]. Thermal pretreatment is an effective sludge disintegration method despite its issue of energy efficiency [22]. The emerging and promising mechanical sludge pretreatment is ultrasonication. Ultrasonication sludge pretreatment has numerous advantages such as efficient sludge disintegration (> 95%), improvement in biodegradability and biosolids quality, no chemical addition, less retention time, sludge reduction and energy recovery [36]. Chemical sludge pretreatments, including acidic, alkaline, and oxidative pretreatments, have also been applied to enhance the sludge biodegradation [17]. Acidic and alkaline treatments are simple and easy to operate. In addition, alkali and acid reagents are effective to solubilize lignin and hemicellulose in the biomass [8, 41]. Microwave pretreatment has also shown good performance [12]. Moreover, pretreatment could also provide sterilization function, which is highly favorable for lipid production from pure culture.

The study aims to recover carbon from sewage sludge by employing oleaginous yeast *Lipomyces starkeyi* for lipid production and simultaneously increase sludge reduction. Acidic pretreatment, alkaline pretreatment, microwave irradiation, and ultrasonication were investigated on the enhancement of nutrients and carbon release from sludge, the increase of lipid production and improvement of sludge reduction. Sludge reduction and reuse for lipid production after pretreatment were compared with other sludge regulation methods and its further application was discussed.

## Results and discussion

### Pretreatment impact on soluble chemical oxygen demand and nutrient release from sludge

Soluble chemical oxygen demand (SCOD) was found to be a reliable character to represent the soluble organics in the liquid phase. The increase of SCOD indicated the release of organic matter from solid into liquid [55].

**Fig. 1:**
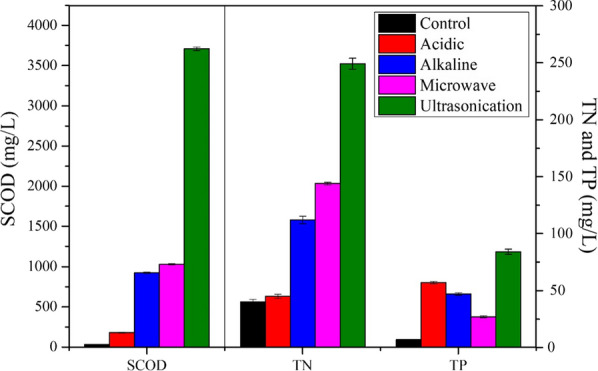
SCOD, TN, and TP variation before and after pretreatments

It can be seen that the SCOD in the supernatant before pretreatment was 32.64 mg/L (Fig. 1). In this study, raw sludge was collected from secondary sedimentation, thus, the SCOD in the supernatant of the raw sludge should be the same as the effluent of secondary sedimentation. It was reported that SCOD in the effluent of secondary sedimentation was generally below 40 mg/L [9, 44]. Hence, the result is consistent with reality.

In this study, it was observed that the SCOD had significantly increased after different pretreatments. The SCOD in the supernatant was increased from 32.64 to 180.25 mg/L, 924.16 mg/L, 1029.89 mg/L, and 3708.31 mg/L after acidic, alkaline, microwave irradiation and ultrasonication pretreatment, respectively (Fig. 1), which was 4.52 times, 26.31 times, 30.55 times and 112.61 times higher than that in the original sludge, respectively. Selvakumar and Sivashanmugam reported that thermo-chemical pretreatment has increase SCOD up to 27.60% [42]. In addition, other researchers have also found that the SCOD increased 7.22 times after thermo-alkaline pretreatment [46]. It suggests that the pretreatments could effectively assist the release of organic matters from solid.

Among all, ultrasonication pretreatment provides the highest SCOD increase which was 2.60, 3.01, and 19.57 times higher than the microwave, alkaline, and acidic pretreatment, respectively (Fig. 1). Therefore, ultrasonication was considered to be the most efficient way for soluble substances release from sludge. During ultrasonication, microbubbles and free radicals are generated which could efficiently destroy microbial cells, breakdown complex organic compounds and release nutrients to the supernatant [36]. It was reported that the release of organic matters and the increase of SCOD with the ultrasonic density and ultrasonic intensity followed the first-order reaction [15, 24, 30, 48]. It has been reported that a neglectable amount of organic matter was oxidized during the ultrasonication pretreatment, and soluble materials were mainly transferred from the solid phase to the liquid phase [20]. In this study, the mixed liquor suspended solids (MLSS) of the sludge solution before and after ultrasonication was 7.14 g/L and 6.72 g/L, respectively. It indicates that the MLSS reduction of sludge due to ultrasonication was 5.88%.

It was noticed that the total nitrogen (TN) in the supernatant of raw sludge was very high (around 40.50 mg/L). The sludge utilized in this study was the secondary sludge collected from secondary sedimentation which is the last unit of wastewater treatment if disinfection is not performed. It means the treated water (called effluent) is discharged into natural water bodies after this unit. The supernatant of the sludge is having the same quality as effluent of the treatment plant as they are the water from the same unit. It was observed that the TN in the supernatant of the sludge was 40.50 mg/L, which indicates that the TN of the effluent was around 40.50 mg/L. According to the Criteria of Grade I of the "Standard for Discharge of Pollutants From Urban Sewage Treatment Facilities" (GB18918-2002), the TN in the effluent should be below 15 mg/L. It indicates that an excessive amount of nitrogen would be discharged to the natural waters if additional treatment has not been applied after secondary treatment. In the last decades, excessive nitrogen discharge from the wastewater treatment plant has caused severe eutrophication and destructed the aquatic ecosystems [53]. Different from other pretreatments, TN concentration in the supernatant was nearly not changed (from 40.50 to 45.00 mg/L) after acidic pretreatment (Fig. 1). It has also been reported that the acidic treatment did not significantly impact the TN concentration in the liquid portion (only 8% increase of the TN concentration after treatment) [47, 49, 50]. Acidic pretreatment could effectively cause cell death due to dehydration; however, it is not efficient for breaking cell membranes [54]. It suggests that no significant release of intracellular protein occurs in acidic pretreatment.

TN in the supernatant was increased from 40.50 to 112.27 mg/L, 143.84 mg/L, and 248.94 mg/L after alkaline, microwave irradiation and ultrasonication pretreatment, respectively (Fig. 1). It can be seen that TN has been released due to pretreatment and the release amount was in the order of ultrasonication > microwave > alkaline > acidic. Both acidic and alkaline treatments are achieved by adjusting solution pH. Compared to acidic pretreatment, alkaline treatment has better performance on TN release. Alkali could react with phospholipid (the main component of the cell membrane) to occur saponification, and thus disrupt the cell and release the intracellular products (such as protein) [23]. It hence increases the TN in the supernatant. It can be seen that microwave and ultrasonication pretreatment achieved efficient TN release and ultrasonication provides better performance (Fig. 1). Microwave radiation provides rapid temperature increase and could efficiently break hydrogen bonds. It leads to the disintegration of proteins and the release of TN [31]. As been discussed above, free radicals were generated during ultrasonication that could break and deconstruct the cells, and thus lead to release of protein and polysaccharide into the supernatant [15, 36].

It was observed that total phosphorus (TP) concentration in the supernatant was significantly increased after acidic pretreatment (from 7.02 to 57.15 mg/L) (Fig. 1). Sludge contains some amount of phosphorus precipitates which would dissociate at low pH conditions. Hence, the TP increase was observed in the liquid phase after acidic pretreatment. As mentioned, cell lysis occurs after alkaline treatment. However, at high pH, PO_4_^3−^ could form precipitate and stay in solid phase. Thus, it leads to the lower TP concentration in the supernatant of alkaline treated sludge compared to that of acidic treatment. As discussed above, microwave is capable of disrupting the complex materials and thus leads to an increase of TP in the supernatant. Among all, ultrasonication was still the best one for releasing TP (from 7.02 to 83.78 mg/L) (Fig. 1).

Overall, ultrasonication was the most efficient method for the release of SCOD, TN and TP. Similar results were reported that SCOD, TP, TN in the supernatant of sludge were significantly increased after ultrasonication pretreatment in which the SCOD, TP, and TN were increased 3.3–5.4, 2.8–4.5, and 13.1–19.6 times after ultrasonication pretreatment [21, 35]. Other pretreatment methods also had some merits on releasing certain nutrients, for instance, acidic pretreatment on the release of TP, alkaline and microwave pretreatment on the release of TN. Further studies could be carried out on the combination of ultrasonication and acidic pretreatment or ultrasonication and alkaline pretreatment to enhance the release of targeted nutrients.

### Sludge pretreatments on lipid production and nutrient utilization

**Fig. 2:**
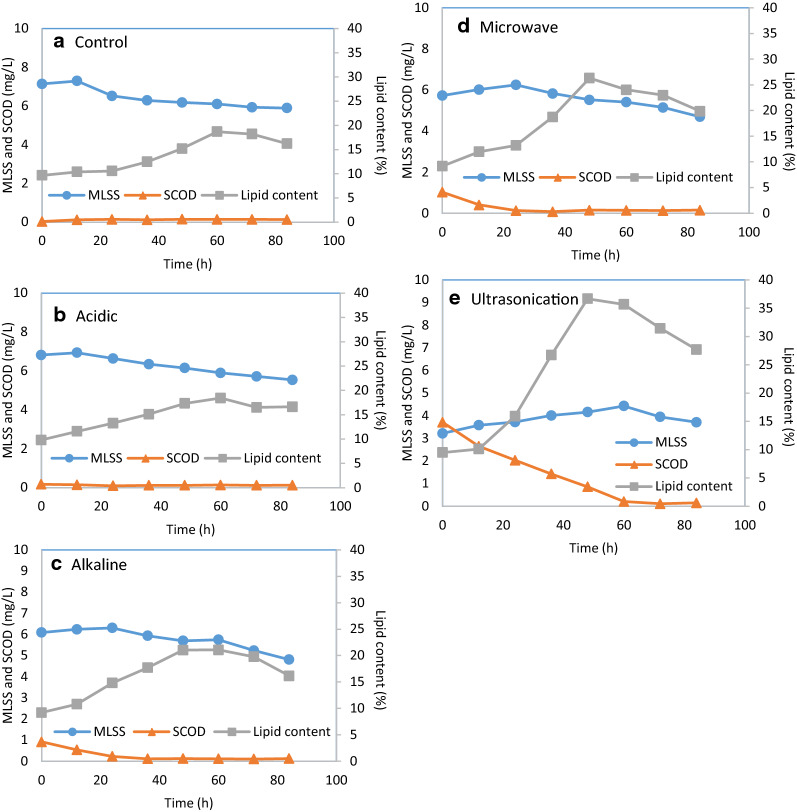
MLSS and lipid content obtained from *Lipomyces starkeyi* cultivated with sludge treated with different method

Due to pretreatment, some suspended solids are dissolved into a soluble form, hence, decrease of MLSS was observed after treatments. The obtained MLSS after sludge pretreatment was the initial MLSS of the fermentation. The results are shown in Fig. 2.

It was observed that the MLSS concentration first increased and then decreased in all the cases (Fig. 2). The increase started after 12 h fermentation in the case of control, acid, and alkaline treated sludge. For microwave and ultrasonication pretreatment cases, the MLSS increase lasted till 24 h and 60 h, respectively. After increasing stage, MLSS gradually decreased in the systems. The increase of MLSS was mainly due to: the inoculation of preculture; the biomass growth by consuming the left substrate from preculture medium; the biomass growth by consuming the SCOD in the medium.

In the fermentation with original sludge, acid or alkaline treated sludge, the available SCOD is very limited (Fig. 1). It is not able to contribute to the biomass growth, hence MLSS increase would be mainly due to the addition of preculture. After the left substrate from the preculture medium is finished, microorganisms start to consume the organic matter in the sludge. The microorganism biomass increases but sludge amount (organic matter decomposition) is decreasing, and the increase is smaller than the decrease as part of the organic matter is emitted in the form of carbon dioxide. Thus, the observed MLSS is in decrease trend. It is similar to aerobic digestion in which significant sludge reduction occurs due to the microorganism growth [26, 43].

In the fermentation with microwave and ultrasonication-treated sludge, the initial SCOD concentrations were high (Fig. 2). During fermentation, the microorganisms consumed SCOD for self-growth which led to the gradual SCOD decrease and MLSS increase. After SCOD was finished, the MLSS started to drop. At the end of the fermentation, the MLSS was in the order of ultrasonication (3.71 g/L) < microwave (4.70 g/L) < alkaline (4.81 g/L) < acid (5.54 g/L) < control (5.89 g/L). It suggests that still great amount of available organic matter remains undegraded in the sludge medium prepared with original sludge, acid, alkaline, and microwave pretreated sludge.

It was observed that lipid content gradually increased until a maximum lipid content was obtained at 48 h (microwave and ultrasonication) or 60 h (control, acid, and alkaline) in the fermentation (Fig. 2). Ultrasonication pretreated sludge medium contained the highest bioavailable materials (SCOD) among all (Fig. 2), and correspondingly the highest lipid content (36.67% g/g) was obtained in the fermentation with ultrasonication pretreatment sludge (Fig. 2). However, it is still largely lower than the reported lipid accumulation potential of the strain (up to 85.10% g/g) [18]. The common explanation was that oleaginous yeast achieved high lipid accumulation in carbon-rich and nitrogen depletion conditions. The carbon source in the raw sludge was not sufficient to support oleaginous yeast to produce high lipid content even after the pretreatments [61]. To achieve high lipid production, the promising solution was to fortify the sludge by mixing sludge with other carbon-rich substrates [57]. Our previous studies proved that the lipid content increased from 35.32% g/g while using solo pretreated sludge medium to 50.13% g/g after addition of crude glycerol to the pretreated sludge [57, 61]. Due to the depletion of substrates, lipid content gradually decreased till the end of the fermentation (Fig. 2). It would be due to the microorganism self-consumption in lipid for supplying energy to cell activities.

It was found that SCOD rapidly dropped during the lipid accumulation period in the fermentation with ultrasonication pretreated sludge, which indicates the fast consumption of substrate by oleaginous yeast (Fig. 2e). Our previous study found that the consumption of substrates in the initial stage was due to the fast cell growth and thereafter was mainly due to lipid production [7]. After 60 h, the depletion of SCOD was observed which caused the fast decrease of lipid content (Fig. 2e) [7].

**Fig. 3:**
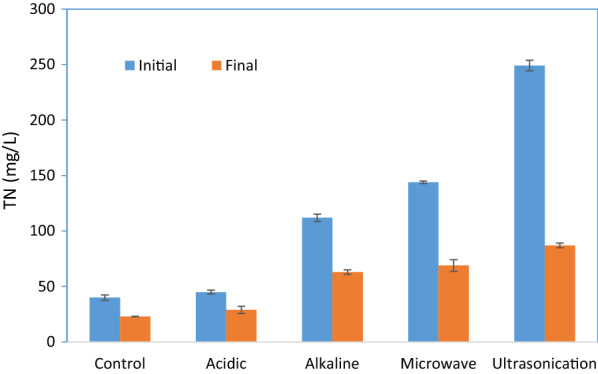
Total nitrogen (TN) concentration at initial and final fermentation

At the end of the fermentations, TN concentration was reduced (Fig. 3). The reduction of nitrogen concentration in the supernatant was owing to the formation of the intracellular material of the strain such as protein. Compared to carbon, nitrogen needed for cell growth was much less [16]. Thus, the utilization amount of nitrogen concentration was less than SCOD amount. The highest TN consumption occurred in fermentation with ultrasonication pretreated sludge (Fig. 3), which is due to better biomass growth in this case compared to others (Fig. 2e).

### Sludge valorization and reduction

**Fig. 4:**
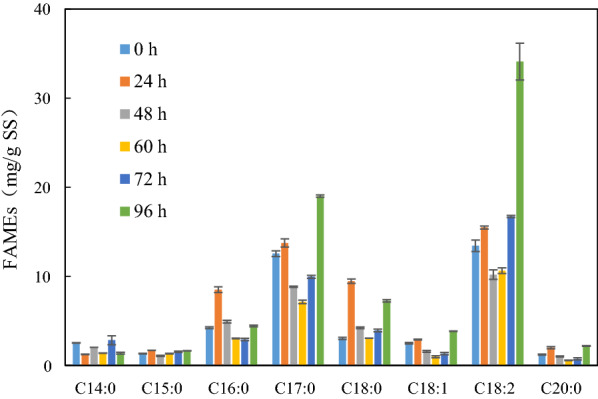
Composition of fatty acid methyl esters (FAMEs)

Ultrasonication pretreated sludge showed the highest lipid production potential, which suggests that it would be the feasible way of biodiesel production from sludge. Lipid extracted from biomass obtained in the fermentation with ultrasonication pretreated sludge was transesterified to biodiesel (fatty acid methyl esters, FAMEs) to evaluate the suitability of the lipid as raw material of biodiesel production. The composition is shown in Fig. 4. It was found that the fractions of C16:0, C17:0, C18:0 and C18:2 continuously increased during the fermentation. Among all, C18:2 was the principal composition (34.10%). The esters with carbon chain of C14–C20 were similar as plant seed oils which is currently used for commercial biodiesel production. Therefore, using ultrasonication pretreated sludge for biodiesel production was applicable.

Sludge reduction is an important target in sludge management. In this study, the sludge reduction due to the lipid production was calculated according to the difference of the initial MLSS of the fermentation and the final solid mass after extraction. The maximum sludge reduction occurred in the fermentation with ultrasonication pretreated sludge which was 63.10%, followed by microwave (43.03%), alkaline (36.44%) and acidic (32.59%).

Ultrasonication and its combination with other pretreatment have also been used for methane and hydrogen production. The sludge reductions in different process were compared (Table 1), and it was found that using ultrasonication pretreated sludge for lipid production achieved remarkable sludge reduction in short time. It was reported that ultrasonication combined with other pretreatment methods had certain advantages on nutrient release and enhancement on sludge reduction [11, 29, 33]. Further study might be performed on the investigation of ultrasonication combined with other pretreatment methods for improving lipid production and sludge reduction.

**Table 1:** Comparison of sludge reduction after ultrasonication pretreatment

Pretreatment	Sludge reduction method	Duration (days)	Sludge reduction (%)	Refs.
Alkaline-ultrasonic pretreatment	Methane production	25	28.68	[11]
Ultrasonic pretreatment	Anaerobic digestion	30	23.7	[28]
Ultrasonic and free nitrous acid pretreatment	Hydrogen production	3	33.6	[33]
Alkaline-ultrasonic pretreatment	Lysis-cryptic growth	12	56.5	[29]
Ultrasonication	Aerobic digestion	3	40.2	[19]
Ultrasonication	Lipid production	2	63.10	This study

## Conclusions

Pretreatment is essential for dissociation of complex materials in sludge. It was found that ultrasonication was more efficient for the release of SCOD, TN and TP compared to acidic, alkaline, and microwave treatment from this study. Compared to the original sludge, the SCOD, TN, and TP increased 112.61 times, 5.22 times, and 11.00 times, respectively, after ultrasonication pretreatment. The highest lipid yield (0.21 g lipid/g dry sludge) and sludge reduction (63.10%) occurred in the fermentation with ultrasonication pretreated sludge. The high release of SCOD from ultrasonication treatment leads to its promising potential as pretreatment of biological sludge management. Combination of ultrasonication with other treatment would provide better performance and related study is demanded.

## Materials and methods

### Materials

#### Sewage sludge

In this study, the raw secondary wastewater sludge was collected from a municipal wastewater treatment plant located in Shenzhen, China. After collection, the sewage sludge was covered and stored at 4 °C. The characterization of the sludge is given in Table 2.

**Table 2:** The characterization of sewage sludge and filtrate

Properties	Unit	Value
pH	–	7.30
Total solids (TS)	g/L	9.40 ± 0.32
MLSS	g/L	7.14 ± 0.69
MLVSS	g/L	4.61 ± 0.22
TN	% TS	2.84 ± 0.10
TP	%TS	4.70 ± 0.25
TN in filtrate	mg/L	40.50 ± 3.43
TP in filtrate	mg/L	7.02 ± 0.30
SCOD in filtrate	mg/L	32.64 ± 5.19

Filtrate was obtained by filtrating sludge with filter paper with pore size 30–50 μm

*MLSS* ^mixed liquor suspended solids, *MLVSS* mixed liquor volatile suspended solids, *TN* total nitrogen, *TP* total phosphorus, *SCOD* soluble chemical oxygen demand^

#### Strain

The lipid-producing strain was oleaginous yeast *Lipomyces starkeyi* which was purchased from China Center of Industrial Culture Collection (CICC). According to the reports, the highest lipid content in *Lipomyces starkeyi* obtained is 85.1% (w/w) [18]. The strain was preserved in 20% (w/w) glycerol at − 80 °C for long-term storage and revival was achieved by streaking onto a potato dextrose agar (PDA) plate [18]. For short-term storage, the strain was maintained in malt extract agar plate and subculture was performed every 7 days.

#### Pre-culture medium

The preculture medium was prepared with Yeast Extract Peptone Dextrose Medium (YPD) (20 g/L glucose, 20 g/L peptone and 10 g/L yeast extract). The pH of the medium was 6.6.

#### Fermenter

In this study, the experiments were carried out in a 5.00 L fermenter (Blbio-5G, Shanghai, China) with a working volume of 3.50 L. The pH, dissolved oxygen (DO), agitation and temperature were automatically controlled during the fermentation. DO was maintained above 30% (v/v) by controlling the agitation (200 rpm—400 rpm) and aeration rate (0.50–3.00 L/min). The temperature was kept at around 28 °C. The pH was not controlled during the whole fermentation as the lipid accumulation only showed slightly difference with and without controlling pH according to shake flask experiment results. Samples (50 mL) were taken at every 12 h during fermentation and stored at 4 °C.

### Experiments

#### Sludge characterization

One liter sludge was directly used to determine MLSS and mixed liquor volatile suspended solids (MLVSS) after transported to lab from the treatment plant.

To analyze MLSS, a quantitative membrane filter paper was dried at 105 ºC until weight was constant. Then, 50 ml of sludge was filtered with the pre-dried filter paper. After filtration, the filtrate was used to determine the TN, TP and SCOD. The filter containing solid was dried at 105 ºC till weight constant. The MLSS was calculated based on Eq. 1.

1 $$ {\text{MLSS}} = {{\left( {M_{2} - M_{1} } \right)} \mathord{\left/ {\vphantom {{\left( {M_{2} - M_{1} } \right)} V}} \right. \kern-\nulldelimiterspace} V}, $$

where *M*_1_ is the weight of membrane filter paper (g); *M*_2_ is the total weight of the dried solid and filter paper (g); *V* is the sample volume, which is 50 mL in this study; the unit of MLSS is g/L.

The filter paper obtained from MLSS analysis was then used for the determination of MLVSS. The filter paper with the dry solid was transferred to a pre-weighed ceramic crucible. Then, the crucible was put in a muffle furnace at 600 °C for 60 min. After cooling down, the crucible was weighed (*M*_4_). MLVSS was calculated according to Eq. 2

2 $$ {\text{MLVSS}} = {{\left[ {\left( {M_{2} - M_{1} } \right) - \left( {M_{4} - M_{3} } \right)} \right]} \mathord{\left/ {\vphantom {{\left[ {\left( {M_{2} - M_{1} } \right) - \left( {M_{4} - M_{3} } \right)} \right]} V}} \right. \kern-\nulldelimiterspace} V}, $$

where *M*_3_ is the weight of the empty ceramic crucible (g); *M*_4_ is the weight of the ceramic crucible with sludge solid after calcination (g).

The property of raw sludge utilized in the study is shown in Table 2.

#### Sludge pretreatment

##### *Acid or base pretreatment* [61]

The 5 mol/L of H_2_SO_4_ solution or NaOH solution was used to adjust the pH of the 4 L sludge solution to 2 or 12. Due to the buffering nature of sludge, several adjustments were performed till the pH was stable at 2 or 12. Then, the sludge solution was stirred for 2 h followed by centrifugation at 25 °C and 5000 rpm (Sigma 3K15, Germany).

##### *Microwave irradiation* [41]

In each run, plate filled with 1 L sludge solution was microwaved for 15 min. During the microwave (900 W) irradiation, the sludge solution was mixed with a 5 min interval. The obtained solutions were well mixed prior to being used for fermentation.

##### *Ultrasonication* [41, 48]

The ultrasonication was achieved by placing the sonication probe in the 2 L beaker containing 0.5 L of sludge solution. The ultrasonication was conducted for 30 min at 450 W and 20 kHz frequency. Ultrasonication was operated with 5 s on and 2 s off mode. The temperature of the sludge was not controlled during ultrasonication. The obtained solutions were employed as medium of oleaginous yeast fermentation.

Sludge samples collected before and after pretreatment were centrifuged and the concentration of SCOD, TN and TP in the supernatant were measured.

#### Fermentation

The preculture was obtained by inoculating a loop full of *Lipomyces starkeyi* to 350 mL sterilized YPD medium and then incubated at 28 ℃ and 150 rpm for 24 h. Then the 350 mL preculture was transferred to the 5 L fermenter with 3.15 L sludge medium. The sludge medium was the whole sludge (solid and liquid) collected from the acidic, alkaline, microwave, or ultrasonication pretreatment after adjusting the pH to around 5.5 with 1 M NaOH or 1 M H_2_SO_4_. The fermentation lasted 84 h and samples (50 mL) were withdrawn every 12 h for analysis.

### Analysis

In this study, biomass referred to the MLSS in the fermentation broth. Thus, it was measured as stated above. To determine lipid [57], a 10 mL sample was centrifuged at 6500 rpm (646,684 g) for 15 min. After discard of the supernatant, the remaining solids were dried at 80 °C for 24 h. The dry solids were transferred to 50 mL solvent-proof tubes. Then, 30 mL of the mixture of chloroform and methanol (2:1 v/v) and 6 mL Zirconia beads (1 mm diameter) were added into the tube and then continuously shaken for 12 h in a wrist action shaker (Burrell Model75). After centrifugation (6500 rpm, 25℃), the bottom layer solution was withdrawn and filtered. The filtrate was put into a pre-weighed glass tube. After evaporation at 80 °C in oven (Hengyi DHG-9145A, Shanghai), the weight difference of the glass tube was the lipid in the 0.01 L sample. Lipid content was calculated according to the following equation:

3 $${\text{Lipid}}\;{\text{content}}\;\left( {\% w/w} \right) = {{{\text{Lipid}}\;{\text{concentration}}} \mathord{\left/ {\vphantom {{{\text{Lipid}}\;{\text{concentration}}} {{\text{biomass}}\;{\text{concentration}}}}} \right. \kern-\nulldelimiterspace} {{\text{biomass}}\;{\text{concentration}}}}.$$

To estimate the potential of the lipid for biodiesel production, the extracted lipid was reacted with a mixture of H_2_SO_4_ and methanol (H_2_SO_4_/CH_4_OH = 1% v/v) at 60 °C for 12 h [57]. The molar ratio of methanol to lipid was set at 6:1. Fatty acid methyl esters (FAMEs, biodiesel) were obtained and extracted with hexane. The composition of biodiesel FAMEs was analyzed using Gas Chromatography linked to Mass Spectroscopy (GC–MS) [58]. The column dimension used was 30 m × 0.25 mm, and a phase thickness was 0.25 μm. The calibration curve was prepared with a mixture comprising 37 FAMEs.

All analysis was duplicated. The results presented in this study were the average value. Standard deviations and Probability (*P* value) of the data obtained in the study were analyzed. The standard deviations are less than 5% and the *P* values were between 0.016 and 0.033 (*P* value < 0.05 means the difference is significant; *P* value < 0.01 means the difference is extremely significant).

## Data Availability

Data sharing not applicable to this article as no datasets were generated or analysed during the current study.
